# Dual Inhibition of CDK4/6 and PI3K/AKT/mTOR Signaling Impairs Energy Metabolism in MPM Cancer Cells

**DOI:** 10.3390/ijms21145165

**Published:** 2020-07-21

**Authors:** Mara Bonelli, Rita Terenziani, Silvia Zoppi, Claudia Fumarola, Silvia La Monica, Daniele Cretella, Roberta Alfieri, Andrea Cavazzoni, Graziana Digiacomo, Maricla Galetti, Pier Giorgio Petronini

**Affiliations:** 1 Department of Medicine and Surgery, University of Parma, 43126 Parma, Italy; mara.bonelli@unipr.it (M.B.); rita.terenziani@unipr.it (R.T.); silvia.zoppi@unipr.it (S.Z.); silvia.lamonica@unipr.it (S.L.M.); daniele.cretella@unipr.it (D.C.); roberta.alfieri@unipr.it (R.A.); andrea.cavazzoni@unipr.it (A.C.); graziana.digiacomo@unipr.it (G.D.); piergiorgio.petronini@unipr.it (P.G.P.); 2 INAIL Research, Department of Occupational and Environmental Medicine, Epidemiology and Hygiene, 00078 Monte Porzio Catone (Rome), Italy; m.galetti@inail.it

**Keywords:** malignant pleural mesothelioma, CDK4/6 inhibition, Palbociclib, PI3K/mTOR inhibitors, metabolism

## Abstract

**Background**: Malignant pleural mesothelioma (MPM) is an aggressive malignancy associated to asbestos exposure. One of the most frequent genetic alteration in MPM patients is *CDKN2A/ARF* loss, leading to aberrant activation of the Rb pathway. In MPM cells, we previously demonstrated the therapeutic efficacy of targeting this signaling with the CDK4/6 inhibitor palbociclib in combination with PI3K/mTOR inhibitors. Here, we investigated whether such combination may have an impact on cell energy metabolism. **Methods**: The study was performed in MPM cells of different histotypes; metabolic analyses were conducted by measuring GLUT-1 expression and glucose uptake/consumption, and by SeaHorse technologies. **Results**: MPM cell models differed for their ability to adapt to metabolic stress conditions, such as glucose starvation and hypoxia. Independently of these differences, combined treatments with palbociclib and PI3K/mTOR inhibitors inhibited cell proliferation more efficaciously than single agents. The drugs alone reduced glucose uptake/consumption as well as glycolysis, and their combination further enhanced these effects under both normoxic and hypoxic conditions. Moreover, the drug combinations significantly impaired mitochondrial respiration as compared with individual treatments. These metabolic effects were mediated by the concomitant inhibition of Rb/E2F/*c*-myc and PI3K/AKT/mTOR signaling. **Conclusions**: Dual blockade of glycolysis and respiration contributes to the anti-tumor efficacy of palbociclib-PI3K/mTOR inhibitors combination.

## 1. Introduction

Malignant pleural mesothelioma (MPM) is a rare malignancy originated from pleura mesothelium, frequently related to asbestos fiber, whose incidence in Europe is expected to peak approximately around 2025 [1]. Only few patients are eligible for the trimodality therapy, including surgery, chemotherapy and radiotherapy, while for the majority of patients the prognosis remains poor and the median survival is around 8–14 months [2]. Genomic analysis of mesothelioma has revealed inactivation of tumor suppressor pathways rather than activation of oncogenes. *BRCA-associated protein 1* (*BAP-1*) and *neurofibromatosis type 2* (*NF2*) mutations have been detected in a high percentage of MPM cases [3,4]. In addition, the *cyclin-dependent kinase inhibitor 2A/alternative reading frame* (*CDKN2A/ARF*) tumor suppressor gene is frequently inactivated in MPM, with a percentage of incidence varying from 50% in the epithelioid subtype to nearly 100% in the sarcomatoid histotype [3,4,5]. *CDKN2A* codes for p16INK^4a^ and its alternate reading frame p14ARF, two cell cycle proteins that negatively regulate the cell cycle progression. In particular, p16INK^4a^ binds to and inhibits CDK4/6 kinases, preventing the association with cyclin D and the subsequent phosphorylation of Rb. By maintaining Rb in a hypo-phosphorylated state, it promotes Rb binding to E2F and leads to G_1_ cell cycle arrest.

Recently, we reported that MPM cancer cells, characterized by the expression of Rb and cyclin D1 and negative for p16INK^4a^, were sensitive to the CDK4/6 inhibitor palbociclib, which induced a cell cycle blockade in the G_0_/G_1_ phase associated with cellular senescence. In addition, we demonstrated that palbociclib induced AKT phosphorylation in MPM cells, confirming previous findings in other cell models [6]. The mechanism underlying the activation of AKT by CDK4/6 inhibitors involves the inhibition of a non-canonical function of Rb. In the cytoplasm, hyper-phosphorylated Rb inhibits the activity of mTORC2 complex by directly binding Sin1, a component of this complex. Therefore, Rb inhibition mediated by CDK4/6 inhibitors results in mTORC2 activation, with consequent induction of AKT, which is a known substrate of mTORC2 [6]. Based on these findings, we combined palbociclib with BEZ235, a dual PI3K and mTORC1-2 inhibitor, or BYL719, a specific inhibitor of the p110α subunit of PI3K, and demonstrated that such combinations enhanced the inhibitory effects on cell proliferation and increased cellular senescence in comparison with single agent treatments [7].

A variety of evidence indicates that the CDK4/6-Cyclin D/Rb/E2F pathway plays a relevant role in the regulation of cell energy metabolism, contributing to the metabolic reprogramming associated with cancer [8]. Along this pathway, the effector E2F contributes to the switch from oxidative to glycolytic metabolism, by inducing the expression of glycolytic enzymes, such as phosphofructokinase, while down-regulating the expression of oxidative genes [9]. In addition, CDK4 and 6 as well as Cyclin D have been demonstrated to control energy metabolism, directly phosphorylating some metabolic enzymes or modulating the activity of metabolic regulators such as AMP-activated protein kinase (AMPK) [10]. Therefore, it is not surprising that the inhibition of the CDK4/6-Cyclin D/Rb/E2F pathway may exert multiple effects on cell energy metabolism [8]. The impact of CDK4/6 inhibitors on cell metabolism has been more extensively studied in estrogen receptor (ER)-positive breast cancer, the only type of cancer in which these drugs have received FDA-approval so far [8].

The PI3K/AKT/mTOR pathway also is a crucial regulator of cell energy metabolism, being involved both in the uptake and in the coordination of glucose fate within the cell. Indeed, AKT induces the expression of a number of glycolytic enzymes, such as hexokinase and phosphofructokinase 1, as well as the expression and recruitment of glucose receptors to the cell membrane [11,12]. In addition, the downstream effector of this pathway mTORC1 regulates cellular metabolism by modulating the expression of a number of proteins, including HIF-1α (involved in glucose import and glycolysis) and sterol regulatory element-binding proteins (SREBPs) (involved in nucleotide biosynthesis and fatty acid metabolism) [13].

Taking into account these aspects, we have extended our previous investigation on palbociclib and PI3K/mTOR inhibitors combination to evaluate its effects on cell energy metabolism in MPM cancer cell lines. In the present study, we demonstrate that the growth-inhibitory effects of the combined therapy with palbociclib and PI3K/mTOR inhibitors are associated with impairment of both glycolysis and mitochondrial respiration in MPM cells, further reinforcing our suggestion that this combination may be a valuable strategy for MPM treatment.

## 2. Results

### 2.1. Metabolic Features of MPM Cell Lines

**Figure 1:**
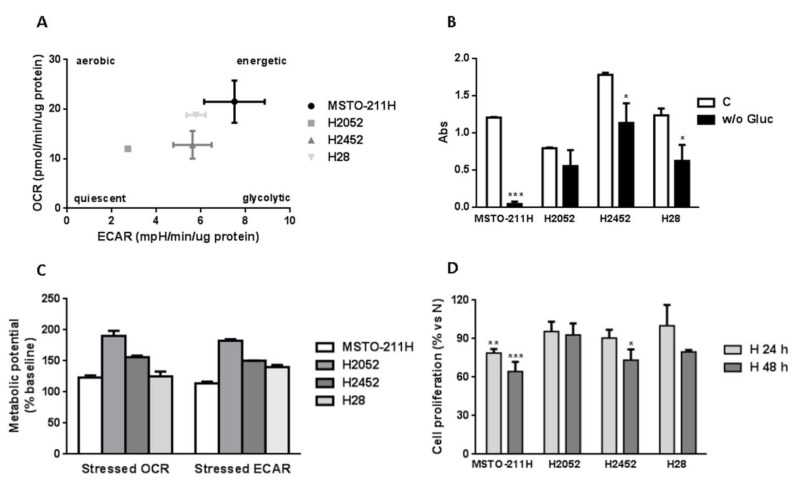
Metabolic features of MPM cell lines. (**A**) The metabolic profile of MSTO-211H, H2052, H2452, and H28 cell lines was defined performing the Agilent SeaHorse Cell energy phenotype assay. After 24 h of growth in complete medium, ECAR and OCR were measured at baseline and after the addition of oligomycin and FCCP. The metabolic potential of each cell line is reported in panel (**C**). Data are representative of two independent experiments. (**B**) MPM cells were maintained for 24 h under glucose starvation (w/o Gluc) and then cell viability was evaluated by CV assay. Data are representative of three independent experiments. * *p* < 0.05, *** *p* < 0.001 vs. control, (**C**). (**D**) MPM cells were grown under hypoxic conditions (1% O_2_) for 24 or 48 h and then cell viability was evaluated by CV assay. Data are expressed as percent versus control cells cultured under normoxic condition (N) and are the media ± SD of three independent experiments. * *p* < 0.05, ** *p* < 0.01, *** *p* <0.001 vs. N.

MPM cell lines of different histotypes (MSTO-211H biphasic, H2452, H28 epithelioid and H2052 sarcomatoid) were analyzed for their metabolic features. As shown in Figure 1A, a seahorse analysis of the cell energy phenotype revealed that MSTO-211H cells were characterized by a pronounced glycolytic and oxidative metabolism, as indicated respectively by high extra cellular acidification (ECAR) and oxygen consumption rate (OCR) levels as compared with the other cell models, and were therefore defined as the most “energetic” cells. On the other hand, H2052 cells were less “energetic”, being less dependent on glycolysis; H28 and H2452 cells had an intermediate behavior. Accordingly, MSTO-211H were highly dependent on glucose for their growth, and 24 h of glucose starvation strongly affected their viability (Figure 1B); H2452 and H28 showed a moderate dependence on glucose. In contrast, H2052 cells were less sensitive to glucose deprivation, presumably because of their greater capacity to adapt to energy stress conditions. Indeed, among these cell models, H2052 cells showed the higher metabolic potential, a measure of cell ability to meet an energy demand via both respiration and glycolysis when exposed to metabolic stressors (Figure 1C). This ability to shift between glycolysis and respiration was supported by the observation that H2052 cells were able to adapt also to hypoxic conditions. As shown in Figure 1D, the proliferation capability of H2052 cells was not affected after 48 h of incubation in hypoxia, whereas the proliferation of MSTO-211H cells, which showed the lower metabolic potential, was significantly reduced already after 24 h of hypoxia exposure.

### 2.2. The Combined Treatment with CDK4/6 and PI3K/mTOR Inhibitors Induces Additive or Synergistic Inhibitory Effects on Cell Proliferation in MPM Cells

**Figure 2:**
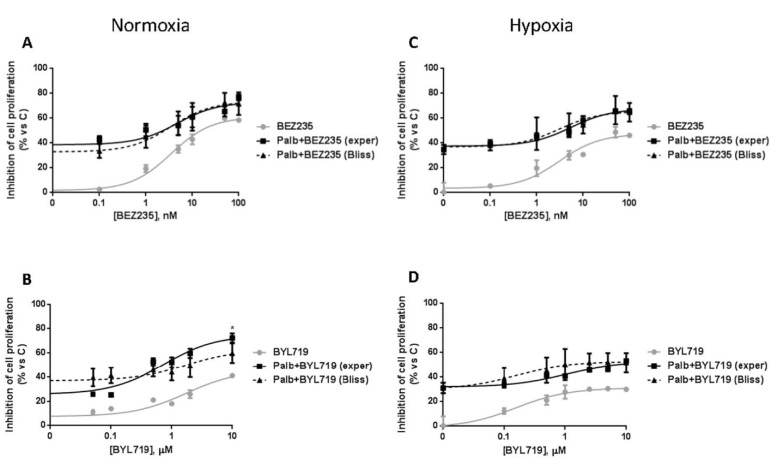
The combined treatment of palbociclib with PI3K/mTOR inhibitors inhibits cell proliferation of H2052 cells under normoxic and hypoxic conditions. H2052 cells were treated with palbociclib 1 µM, combined with increasing concentrations of BEZ235 (from 0.1 nM to 100 nM) (**A**,**C**) or BYL719 (from 0.1 to 10 µM) (**B**,**D**) for 72 h under normoxic (**A**,**B**) or hypoxic conditions (**C**,**D**). Cell proliferation was assessed by MTT assay. The type of interaction (antagonistic, additive or synergistic) was evaluated through Bliss analysis. Data are expressed as percent inhibition versus control cells and are representative of two independent experiments. * *p* < 0.05 vs. Bliss.

We previously demonstrated that the MPM cell lines here analyzed were sensitive to palbociclib treatment, with IC_50_ values ranging from 0.1 to 1.2 μM [7]. The combination of palbociclib with the PI3K/mTOR inhibitors BEZ235 and BYL719 in MSTO-211H and H28 cells and in ZS-LP primary culture cells induced a synergistic or additive inhibition of cell proliferation, depending on both the cell model analyzed and the schedule of drug combination used [7]. Similar results were obtained in H2052 cells: as shown in Figure 2A,B, simultaneous treatments with palbociclib and BEZ235 or BYL719 produced an additive inhibition of cell proliferation, confirming their efficacy for MPM treatment. In addition, we demonstrated that these drug combinations were more effective than single agents in inhibiting H2052 cell proliferation also under hypoxic conditions (Figure 2C,D). This aspect is of particular relevance considering that hypoxia is frequently associated with tumor growth and progression, and hypoxic areas are frequently found in MPM. The efficacy of the drug combinations under hypoxia may acquire further importance for those tumor cells, like H2052 cells, that are more resistant to hypoxia due to their ability to adapt to energetic stress conditions.

### 2.3. The Combined Treatment with CDK4/6 and PI3K/mTOR Inhibitors Impairs Glucose Metabolism in MPM Cells

**Figure 3:**
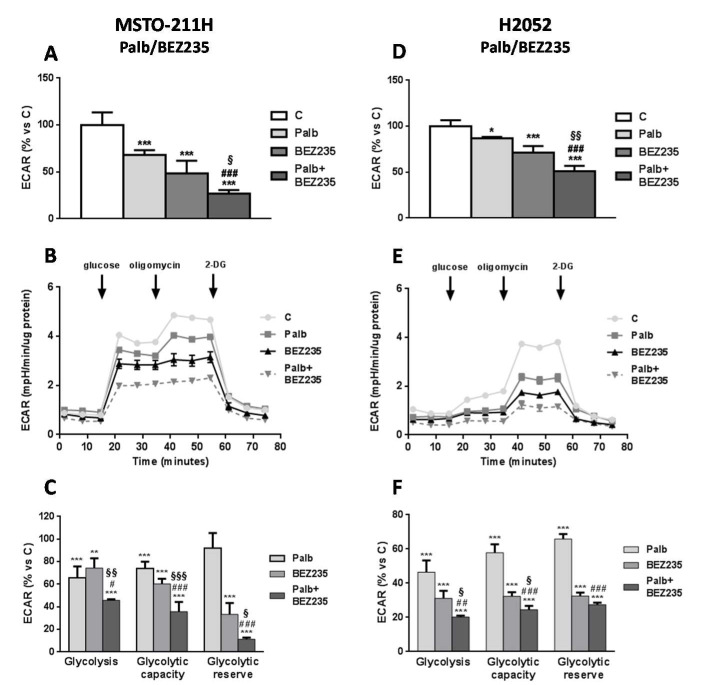
The combined treatment of palbociclib with BEZ235 impairs the glycolytic activity of MSTO-211H and H2052 MPM cells. (**A**,**D**) The glycolytic activity was measured using the Agilent SeaHorse XP analyzer after 24 h of treatment with palbociclib 1 µM, BEZ235 40 nM, or the combination of palbociclib with BEZ235. ECAR data are shown as mpH/min normalized to proteins and represent the mean ± SD of *n* = 4 independent measurements under normal cell culture conditions. (**B**,**E**) Glycolytic profiles were obtained using the Agilent SeaHorse Glycolysis Stress Test. MSTO-211H and H2052 cells were treated as described above. The ECAR was measured under glucose starvation and after the sequential addiction of glucose (substrate for glycolysis), oligomycin (added to maximize the glycolytic flux of the cell) and 2-DG (added to shut down the glycolytic process). The ECAR profiles are representative of two different experiments that yielded similar results. (**C**,**F**) The parameters of glycolysis, glycolytic capacity, and glycolytic reserve were calculated as percent versus control cells. The data are expressed as the media ± SD of at least three independent measurements. * *p* < 0.05, ** *p* < 0.01, *** *p* < 0.001 vs. control; ^#^ *p* < 0.05, ^##^ *p* < 0.01, ^###^ *p* < 0.001 vs. palbociclib; ^§^ *p* < 0.05, ^§§^ *p* < 0.01, ^§§§^ *p* < 0.001 vs. BEZ235.

A variety of evidence indicates that cell cycle-related proteins are directly involved in the regulation of cell energy metabolism [9]. Therefore, we sought to evaluate whether the growth-inhibitory effects of the combined treatment with palbociclib and PI3K/mTOR inhibitors were associated with alterations of cell energy metabolism. This study was performed in MSTO-211H and H2052 cells, defined as the most and the less “energetic” cells, respectively, among the MPM cell models analyzed (crf Figure 1A). The glycolytic activity of MSTO-211H and H2052 cells was assessed following real-time changes in ECAR levels. As shown in Figure 3A,D, a 24 h treatment with either palbociclib or BEZ235 reduced the basal ECAR levels in both cell models. Interestingly, the combination of palbociclib with BEZ235 further down-regulated glycolysis in comparison with single drug treatments.

Then, the impact of the drug treatments on cell glycolytic activity was more deeply investigated by performing the Agilent SeaHorse Glycolysis Stress Test. As shown in Figure 3B–F, palbociclib combined with BEZ235 was more effective than single agents in preventing the increase of the glycolytic rate induced by glucose addition in both MSTO-211H and H2052 cells. Moreover, the maximum glycolytic capacity, measured as the maximum ECAR rate achieved upon inhibition of oxidative phosphorylation (OXPHOS) by oligomycin, was significantly down-regulated by either palbociclib or BEZ235 and even more by their combination. The glycolytic reserve, which measures the capability of cells to respond to an energetic demand, was also strongly hindered by the combined treatment.

**Figure 4:**
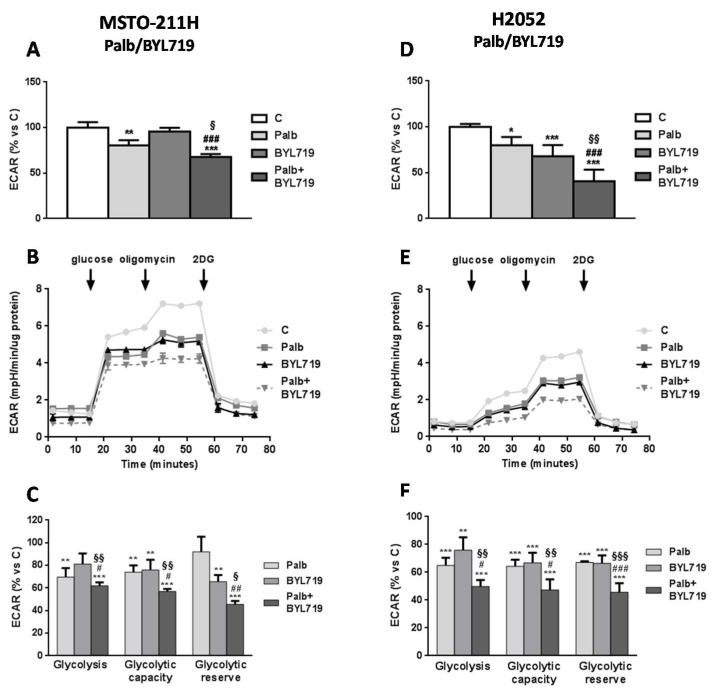
The combined treatment of palbociclib with BYL719 impairs the glycolytic activity of MSTO-211H and H2052 MPM cells. (**A**,**D**) The glycolytic activity was measured using the Agilent SeaHorse XP analyzer after 24 h of treatment with palbociclib 1 µM, BYL719 (1 µM for MSTO-211H cells or 2.5 μM for H2052 cells) or the combination of palbociclib with BYL719. ECAR data are shown as mpH/min normalized to proteins and represent the mean ± SD of *n* = 4 independent measurements under normal cell culture conditions. (**B**,**E**) Glycolytic profiles were obtained using the Agilent SeaHorse Glycolysis Stress Test; the ECAR was measured under glucose starvation and after the sequential addiction of glucose, oligomycin and 2-DG. The ECAR profiles are representative of two different experiments that yielded similar results. (**C**,**F**) The parameters of glycolysis, glycolytic capacity, and glycolytic reserve were calculated as percent versus control cells. The data are expressed as the media ± SD of at least three independent measurements. ** *p* < 0.01, *** *p* < 0.001 vs. control; ^#^ *p* < 0.05, ^##^ *p* < 0.01, ^###^ *p* < 0.001 vs. palbociclib; ^§^ *p* < 0.05, ^§§^ *p* < 0.01, ^§§§^ *p* < 0.001 vs. BYL719.

The impact on glycolytic activity was then evaluated combining palbociclib with BYL719. Differently from BEZ235, BYL719 treatment alone reduced glycolysis only in H2052 cells (Figure 4A,D). However, when the cells were treated simultaneously with palbociclib and BYL719, glycolysis as well as the glycolytic capacity and reserve were strongly down-regulated as compared with single drug treatments in both cell models (Figure 4B–F).

**Figure 5:**
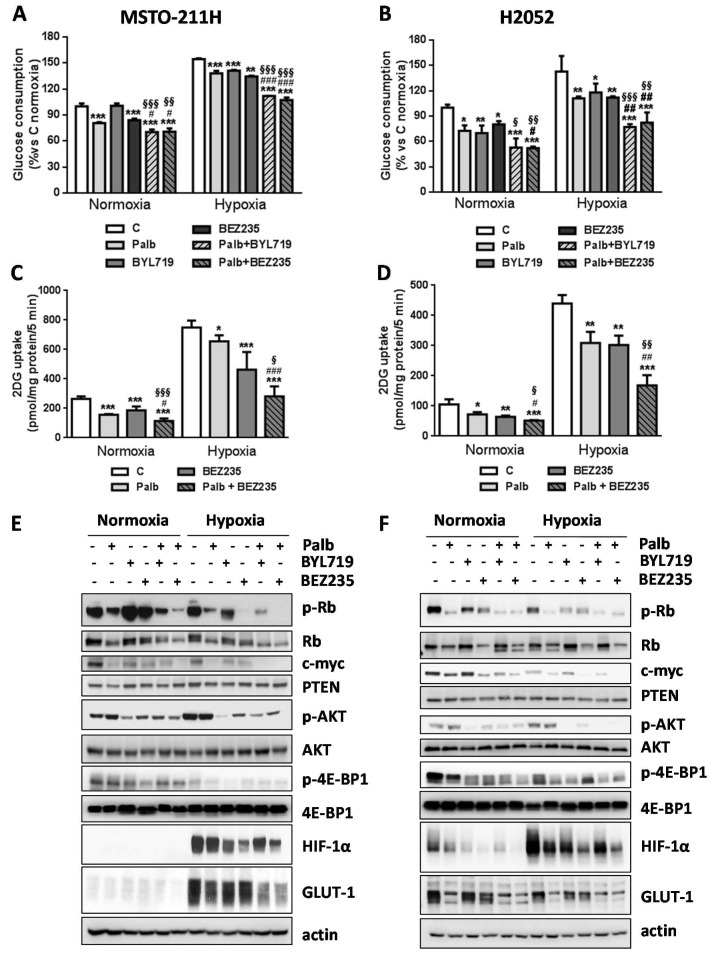
The combined treatment of palbociclib with PI3K/mTOR inhibitors hinders energy metabolism by down-regulating Rb/E2F/*c*-myc and PI3K/AKT/mTOR signaling in MSTO-211H and H2052 MPM cells. (**A**,**B**) Cells were treated with palbociclib 1 µM, BEZ235 40 nM, BYL719 (1 µM for MSTO-211H cells or 2.5 μM for H2052 cells) or the combination of palbociclib with BEZ235 or BYL719 for 24 hunder normoxic or hypoxic (1% O_2_) conditions for 24 h. At the end of the treatments, glucose consumption was measured. * *p* < 0.05,** *p* < 0.01,*** *p* < 0.001 vs. C normoxia; ^#^ *p* < 0.05, ^##^ *p* < 0.01, ^###^ *p* < 0.001 vs. palbociclib; ^§^ *p* < 0.05, ^§§^ *p* < 0.01, ^§§§^ *p* < 0.001 vs. BEZ235 or BYL719. (**C**,**D**) Cells were treated with palbociclib 1 µM or BEZ235 40 nM alone or in combination under normoxic or hypoxic (1% O_2_) conditions for 24 h. At the end of the treatment, glucose uptake was measured. * *p* < 0.05, ** *p* < 0.01, *** *p* < 0.001 vs. C; ^#^ *p* < 0.05, ^##^ *p* < 0.01, ^###^ *p* < 0.01 vs. palbociclib; ^§^ *p* < 0.05, ^§§^ *p* < 0.01, ^§§§^ *p* < 0.01 vs. BEZ235. MSTO-211H (**E**) and H2052 (**F**) cells were treated as in (**A**,**B**), then Western blotting analysis was performed to evaluate the phosphorylation/expression of proteins involved in cell cycle regulation, PI3K/AKT/mTOR signaling, and glucose metabolism. The results are representative of two independent experiments.

The inhibitory effects of palbociclib and PI3K/mTOR inhibitors on glycolysis reflected a reduction of glucose consumption in both MSTO-211H and H2052 cells (Figure 5A,B). In particular, under normoxic conditions, treatment with either palbociclib or BEZ235 alone induced ~20% decrease in glucose consumption, while BYL719 alone had effect only in H2052 cells. Importantly, a stronger decrease (~30% and 40% in MSTO-211H and H2052 cells, respectively) was promoted by the combination of palbociclib with both PI3K/mTOR inhibitors, confirming its negative impact on glucose utilization. Interestingly, when exposed to hypoxic conditions, the cells increased their glucose utilization; the drug combination hindered hypoxia-mediated stimulation of glucose consumption more efficaciously than individual treatments (30% decrease vs. 15–20% after single drug treatments). The reduced glucose consumption promoted by the drug treatments correlated with the down-regulation of glucose uptake. Indeed, palbociclib and BEZ235 as single agents reduced the glucose uptake and the combination enhanced this effect under both normoxic and hypoxic conditions (Figure 5C,D). As previously observed for glucose consumption, the drug combination significantly inhibited the adaptive increase of glucose uptake promoted by hypoxia.

As shown in Figure 5E,F, the greater efficacy of palbociclib-PI3K/mTOR inhibitors combined treatment was presumably ascribed to the simultaneous inhibition of *c*-myc and PI3K/AKT/mTOR signaling, both known to be involved in the modulation of glucose metabolism [14].

In MSTO-211H cells, palbociclib-dependent down-regulation of Rb phosphorylation was potentiated by the combination with either BEZ235 or BYL719, resulting in a stronger decreased expression of *c*-myc, a direct target of the E2F transcription factor (more evident under hypoxic conditions). In H2052 cells, both palbociclib and PI3K/mTOR inhibitors alone reduced Rb phosphorylation, although no further down-regulation was promoted by the combinations; however, being *c*-myc expression modulated also by mTORC1, the concomitant inhibition of Rb and mTORC1 by the combinations produced a stronger down-regulation of *c*-myc also in these cells.

On the other hand, BEZ235 and BYL719 significantly reduced basal and palbociclib-mediated induction of AKT phosphorylation/activation, leading to down-regulation of mTORC1 signaling, as demonstrated by the reduced activity of its downstream target 4E-BP1. These effects were observed under both normoxic and hypoxic conditions. It is worth noting that both MSTO-211H and H2052 cells were positive for PTEN protein (a negative regulator of AKT activation frequently lost in tumors including MPM [15]) and its expression was not affected by the drug treatments.

In MSTO-211H cells, hypoxia induced the stabilization and accumulation of HIF-1α and up-regulated the expression of GLUT-1. GLUT-1 protein levels were decreased by palbociclib and the PI3K/mTOR inhibitors alone and even more by the drug combinations; in contrast, only the PI3K/mTOR inhibitors were able to reduce HIF-1α accumulation, an effect that might contribute to reduce GLUT-1 and hence glucose utilization under hypoxia.

In H2052 cells, GLUT-1 was detectable also under normoxia and was not increased by hypoxia. In addition, its expression levels were strongly reduced by palbociclib treatment, although only a slight further decrease was promoted by the simultaneous treatment with PI3K/mTOR inhibitors. Therefore, in H2052 cells down-regulation of glucose transporters other than GLUT-1 might be involved in the inhibitory effects on glucose metabolism promoted by the drug combinations.

Altogether, these findings suggest that targeting glucose metabolism through the combined inhibition of the Rb/E2F/*c*-myc axis and the PI3K/AKT/mTOR signaling may contribute to the anti-tumor efficacy of palbociclib-PI3K/mTOR inhibitors combinations in MPM cells.

### 2.4. The Combined Treatment with CDK4/6 and PI3K/mTOR Inhibitors Inhibits Mitochondrial Respiration in MPM Cells

**Figure 6:**
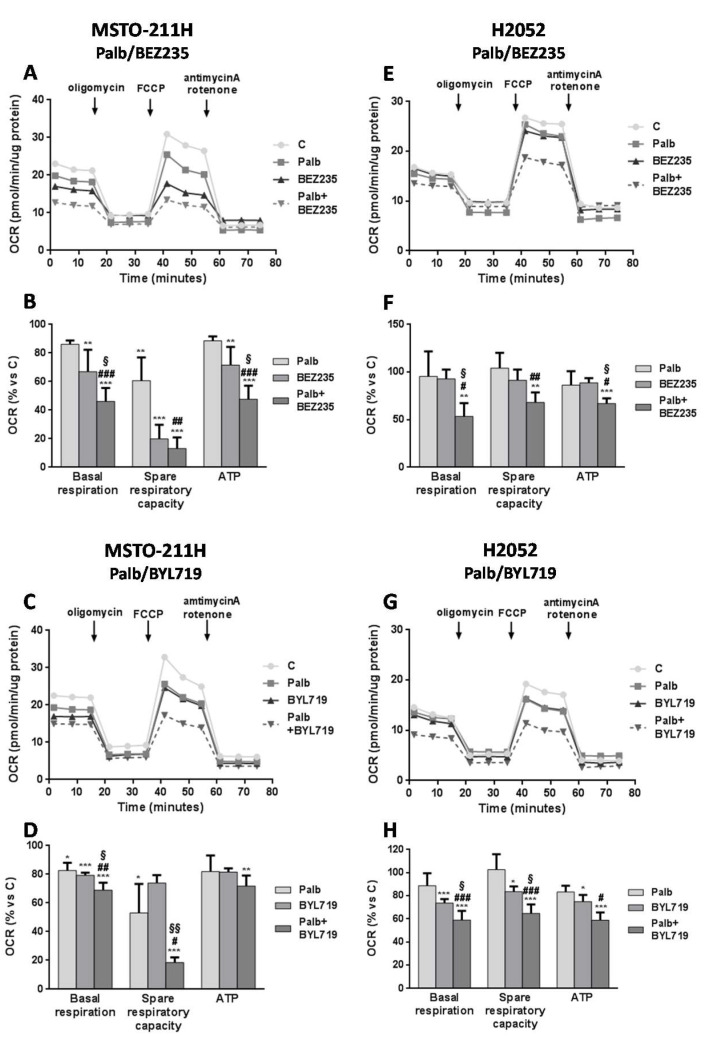
The combined treatment of palbociclib with PI3K/mTOR inhibitors impairs mitochondrial respiration in MSTO-211H and H2052 MPM cells. (**A**,**C**,**E**,**G**) Mitochondrial respiration profiles were analyzed by the Agilent SeaHorse Cell Mito Stress Test. MSTO-211H and H2052 cells were treated with palbociclib 1 µM, BEZ235 40 nM, BYL719 (1 µM for MSTO-211H cells or 2.5 μM for H2052 cells) or the combination of palbociclib with BEZ235 or BYL719 for 24 h. The OCR data are shown as mpH/min normalized to proteins and the OCR profiles are representative of two independent experiments. OCR was measured in normal culture conditions and after the sequential addiction of oligomycin (an ATP-synthase inhibitor), FCCP (an uncoupling agent that disrupts the mitochondrial membrane potential and maximizes OCR) and a mixture of antimycin A/rotenone, which shuts down mitochondrial respiration by inhibiting the electron transport chain. (**B**,**D**,**F**,**H**) The parameters of basal respiration, spare respiratory capacity, and ATP production were calculated as percent versus control cells. The results are expressed as the media ± SD of at least three independent measurements. * *p* < 0.05, ** *p* < 0.01, *** *p* < 0.001 vs. control; ^#^ *p* < 0.05, ^##^ *p* < 0.01, ^###^ *p* < 0.001 vs. palbociclib; ^§^ *p* < 0.01, ^§§^ *p* < 0.001 vs. BEZ235 or BYL719.

Considering that both mTORC1 and *c*-myc have a well-recognized role not only in the modulation of glucose metabolism, but also in the regulation of mitochondrial function [16,17], we investigated the impact of palbociclib plus PI3K/mTOR inhibitors on mitochondrial respiration. Treatment of MSTO-211H (Figure 6A,B) or H2052 (Figure 6C,D) cells with palbociclib plus BEZ235 significantly down-regulated the basal respiration, calculated by subtracting the non-mitochondrial oxygen consumption to the baseline OCR. This combination impaired also the energetic flexibility of the cells, reducing their spare respiratory capacity; this parameter was calculated by exposing the cells to FCCP, which mimics a physiological energy demand stimulating respiration to its maximum capacity. In addition, the respiration associated with ATP production was significantly decreased by the combination in comparison with single agent treatments. Likewise, the combination of palbociclib with BYL719 impaired the mitochondrial function (Figure 6E–H).

## 3. Discussion

The metabolic shift of cancer cells towards glycolysis, known as Warburg effect, is a hallmark of cancer and MPM cells are not an exception to this rule, as demonstrated by the high glycolytic rate found in MPM lesions, which renders these tumors usually positive to positron emission tomography with 2-deoxy-2-[fluorine-18]fluoro-D-glucose (18F-FDG PET) [18,19].

Among the mechanisms involved in the Warburg effect, activation of PI3K/AKT/mTOR and Rb/E2F/myc signaling pathways plays a relevant role [12].

In the contest of MPM, it has been demonstrated that the PI3K/AKT/mTOR pathway is frequently up-regulated probably because of the high release of reactive oxygen species (ROS) resulting from asbestos exposure. Indeed, ROS can directly lead to the inactivation of PTEN, a lipid phosphatase involved in the negative regulation of this pathway, through the oxidation of its cysteine residues [20]. Recently, loss of PTEN expression has been reported in a high percentage of human sarcomatoid MPM as compared with other subtypes [15]. In addition, components of the PI3K/AKT/mTOR pathway have been found to be deregulated in MPM, in particular mutations in the catalytic subunit of PI3K have been frequently detected in MPM patients and associated to disease progression [21,22]. Interestingly, mTOR activity is aberrantly up-regulated in neurofibromatosis type 2 (NF2)-inactivated tumors [23], suggesting that this pathway might strongly affect glucose metabolism in H2052 cells, reported as NF2 mutated [24]. Indeed mTORC1, together with other components of the PI3K/AKT/mTOR pathway, modulates the expression of a number of proteins involved in glucose import and glycolysis, contributing to cancer metabolic reprogramming [13].

Activation of the Rb/E2F/myc axis also has been frequently found in MPM, as a consequence of the high incidence of *CDKN2A/ARF* loss [3,25]. As a result, the Rb protein is continuously hyper-phosphorylated by the CDK4/6-Cyclin D complexes, thus releasing the E2F transcription factor, which can exert its action on the modulation of glucose metabolism. In addition, downstream of E2F, *c*-myc transcription factor may directly contribute to the Warburg effect promoting the expression of glycolytic genes such as phosphofructokinase 1, hexokinase, enolase, and LDH as well as glucose transporters [26,27].

In a previous paper, we demonstrated that *CDKN2A/ARF* gene alterations might represent a new attractive target for MPM treatment and found that CDK4/6 inhibition by palbociclib in combination with PI3K/mTOR inhibitors strongly reduced cell proliferation and induced cellular senescence [7]. In the present study, we demonstrated the efficacy of such combinations in the impairment of glucose metabolism in MPM cell lines.

Firstly, we found that MPM cell lines are characterized by different metabolic features. Interestingly, H2052 cells, despite showing the less “energetic” phenotype among the MPM cell models analyzed, demonstrated a superior metabolic plasticity, being able to shift towards glycolysis or mitochondrial respiration depending on the energy stress imposed; this resulted in a superior capacity to adapt to adverse environment conditions, such as hypoxia or glucose deprivation.

We found that palbociclib and PI3K/mTOR inhibitors were able to hinder glucose metabolism in MPM cells when given alone, and the combined drug treatments enhanced these metabolic effects. In particular, the combined treatments significantly reduced glycolysis, glycolytic capacity and reserve (measured as ECAR) more efficaciously than single drug treatments, suggesting their efficacy in preventing the cell adaptation to enhanced energy demands under stressful conditions. Accordingly, glucose consumption and uptake were more strongly down-regulated by the combinations and this effect was observed also under hypoxic conditions. These results corroborate our previous findings in other cancer models [28]. Interestingly, impairment of glycolysis promoted by the combined treatments was not related to the baseline metabolic phenotype of the cells. Indeed, H2052 and MSTO-211H cells were similarly affected, despite their different ability to deal with metabolic stress. In this regard, it is worth noting that MSTO-211H cells exposed to hypoxia increased glucose uptake and consumption as H2052 cells did, but failed to efficiently adapt to this stress condition, reducing their proliferation already after 24 h.

Mechanistically, the drug treatments had different effects on the signaling transduction pathways involved in the regulation of energy metabolism depending on the cell model analyzed. In both cell lines, we demonstrated that palbociclib hindered the glycolytic process through the inhibition of the Rb/E2F/*c*-myc axis. This result is in line with previous observations in triple negative breast cancer cells (TNBC) [28,29]. In H2052 cells, palbociclib inhibited also HIF-1α expression and prevented its accumulation under hypoxia, confirming previous findings showing that palbociclib destabilizes HIF-1α in colon cancer cells [30]. In addition, c-myc is known to cooperate with HIF-1 to induce the expression of glycolytic enzymes [31], a mechanism whose inhibition may further contribute to palbociclib-mediated impairment of glucose utilization. The PI3K/AKT/mTOR pathway also regulates HIF-1α expression, and treatment with PI3K/mTOR inhibitors strongly inhibited the expression of this protein in both MPM cell lines. In addition to this mechanism, inhibition of AKT and mTOR might directly contribute to hinder glucose utilization, considering their role in controlling glucose metabolism at multiple levels, as previously mentioned. A correlation between the PI3K/AKT/mTOR pathway and glucose metabolism in MPM has been described by Kaira and collaborators, who demonstrated that inhibition of mTORC1/C2 as well as HIF-1α decreased 18F-FDG uptake in MPM cell lines [32].

In both MPM cell models, the concomitant inhibition of Rb/E2F/*c*-myc and PI3K/AKT/mTOR pathways resulted in a stronger down-regulation of glucose metabolism as compared with single agents, providing a mechanism that contributed to the superior efficacy of the drug combinations.

In addition to these effects on glycolysis, we demonstrated that the combined treatment induced a consistent and significant inhibitory effect on OCR, indicative of a dysfunction of mitochondrial oxidative metabolism. In particular, basal respiration, spare respiratory capacity, and ATP production were strongly impaired after the exposure to the drug combinations in both cell lines. The stronger down-regulation of *c*-myc may account for these enhanced inhibitory effects. Indeed, it is well established that *c*-myc exerts a central role in mitochondrial biogenesis, driving the expression of several nuclear-encoded mitochondrial genes as well as mitochondrial genes, including those encoding for cytochrome c, a key regulator of mitochondrial respiration, and cytochrome oxidase 5b, a subunit of Complex IV of the electron transport chain [33]. However, a contribution to the significant impairment of mitochondrial function by the drug combinations might also come from the inhibition of the PI3K/AKT/mTOR pathway, which also converges on the regulation of mitochondrial respiration. In particular, mTORC1 is known to coordinate mitochondrial energy production by stimulating the synthesis of nuclear-encoded mitochondrial genes, including TFAM, mitochondrial ribosomal proteins and components of complexes I and V of the electron transport chain [34]. mTORC1-mediated control of mitochondrial activity has been demonstrated to occur through 4E-BP1-dependent translational regulation, and mTORC1 inhibition has been shown to strongly decrease mitochondrial biogenesis and respiration in 4E-BP1 proficient cells [35].

Dual blockade of glycolysis and OXPHOS by palbociclib and PI3K/mTOR inhibitors may be of particular relevance for targeting MPM cancer cells with enhanced metabolic plasticity, which can better adapt to metabolic stress by up-regulating alternative routes when a specific metabolic pathway is inhibited. Accordingly, previous studies demonstrated that combining glycolysis inhibitors (2-DG or GNE-140) with OXPHOS inhibitors (phenformin or metformin) halts cancer cell growth in vitro and in mouse xenograft models by hindering the metabolic reprogramming that could allow cancer cells to survive if only a single metabolic pathway was targeted [36].

In conclusion, this study reinforces our previous suggestion that the combination of palbociclib with PI3K/mTOR inhibitors may represent a valuable therapeutic strategy for MPM treatment, highlighting its efficacy as a novel approach for targeting distinct metabolic features of MPM cancer cells.

## 4. Materials and Methods

### 4.1. Cell Culture

Human MPM cell lines MSTO-211H (biphasic histotype), H2452, H28 (epithelioid histotype), H2052 (sarcomatoid histotype) were cultured in RPMI supplemented with 2 mM glutamine, 10% fetal bovine serum (FBS), and 100 U/mL penicillin/100 μg/mL streptomycin and maintained at 37 °C in a humified atmosphere containing 5% CO_2_. The cells were purchased by the American Type Culture Collection ATCC (Manassas, VA, USA), which authenticates the phenotypes of these cell lines on a regular basis. Hypoxic conditions were established by placing the cells in a tissue culture incubator with controlled O_2_ levels (Binder GmbH, Tuttlingen, Germany).

### 4.2. Drug Treatment

Palbociclib (PD-0332991), BEZ235 and BYL719 were provided by Selleckchem (Houston, TX, USA). Palbociclib was dissolved in sterile water; BEZ235 and BYL719 were prepared in DMSO, and DMSO concentration never exceeded 0.1% (*v/v*); equal amounts of the solvent were added to control cells.

### 4.3. Analysis of Cell Proliferation

Cell viability/proliferation was assessed by cell counting in a Burker hemocytometer with trypan blue exclusion method and by Crystal Violet (CV) or MTT assay, as previously described [37]. The nature of the interaction between palbociclib and PI3K/mTOR inhibitors was calculated using the Bliss additivism model [38]. A theoretical dose-response curve was calculated for combined inhibition using the equation of Bliss = EA + EB − EA * EB, where EA and EB are the percent of inhibition versus control obtained by BYL719 or BEZ235 (A) and palbociclib (B) alone and the E Bliss is the percent of inhibition that would be expected if the combination was exactly additive. If the combination effect is higher than the expected Bliss equation value, the interaction is synergistic, while if the effect is lower, the interaction is antagonistic. Otherwise, the effect is additive and there is no interaction between the drugs.

### 4.4. Western Blotting

Protein extraction, solubilization, and protein analysis by Western blotting were performed as described [39]. Antibodies against p-Rb^Ser780^, Rb, *c*-myc, PTEN, p-AKT^Ser473^, AKT, p-4E-BP1^Thr37/46^, 4E-BP1 were from CST (Danvers, MA, USA); anti-GLUT-1 was from Abcam (Cambridge, UK). The antibody against HIF-1α was from BD Biosciences (Franklin Lakes, NJ, USA). Anti-β-actin was from BioVision (Milpitas, CA, USA). The procedure for GLUT-1 detection was previously described [40]. Horseradish peroxidase-conjugated secondary antibodies and chemiluminescence system were from Millipore (Millipore, MA, USA). Reagents for electrophoresis and blotting analysis were from BIO-RAD Laboratories (Hercules, CA, USA). The chemiluminescent signal was acquired by C-DiGit^®^. Blot Scanner and the spots were quantified by Image Studio^TM^ Software, LI-COR Biotechnology (Lincoln, NE, USA).

### 4.5. Metabolic Assays

#### 4.5.1. Glucose Uptake and Consumption

Glucose uptake was measured using the non metabolizable analogue Deoxy-D-glucose-2-[1,2-3H(N)] (2-DG, PerkinElmer, Waltham, MA, USA) and expressed as pmol of 2-DG/mg protein/5 min, as described previously [40]. Glucose consumption was determined by using a Glucose (HK) Assay Kit (Sigma-Aldrich, St. Louis, MO, USA) and calculated by subtracting the glucose amount in the spent media to glucose in cell-free media. Data were calculated as mg glucose/mg protein and expressed as percent vs. control.

#### 4.5.2. OCR and ECAR Measurements

The oxygen consumption rate (OCR) and extracellular acidification rate (ECAR) were measured using a Seahorse Extracellular Flux XFp analyzer (Seahorse Bioscience, North Billerica, MA, USA) according to the manufacturer’s instructions. OCR and ECAR rates are indicators of mitochondrial respiration and glycolysis, respectively.

The metabolic cell phenotype analysis was conducted using Agilent Seahorse XFp Cell Energy Phenotype Test kit, by measuring mitochondrial respiration and glycolysis under baseline (in the presence of non-limiting quantity of substrates) and stressed conditions (induced by the simultaneous injection of oligomycin, an inhibitor of ATP synthase, and FCCP, a mitochondrial uncoupling agent). The metabolic potential is the measure of cells’ ability to meet an energy demand via respiration and glycolysis, and is calculated as percent increase of stressed OCR over baseline OCR, and stressed ECAR over baseline ECAR.

The glycolytic activity of the cells was measured by evaluating the ECAR value at baseline culture conditions and more deeply by using the Agilent Seahorse XFp Glycolysis Stress Test, which measured ECAR after the sequential injection of glucose, oligomycin and 2-deoxy-glucose (2-DG). With this test, baseline glycolysis was measured as the ECAR rate reached by previously deprived-glucose cells after the addition of saturating amounts of glucose. The glycolytic capacity was the maximum ECAR rate reached by the cells following the addition of oligomycin, which shut down oxidative phosphorylation and drove the cells to use glycolysis to their maximum capacity. Finally, the glycolytic reserve indicated the capability of a cell to respond to an energetic demand and was calculated as the difference between glycolytic capacity and glycolysis.

The mitochondrial respiration was analyzed using Seahorse XFp Analyzer Mito Stress kit. Baseline respiration levels were measured by subtracting non-mitochondrial respiration (OCR values obtained after injection of antimycin A/rotenone) from initial OCR levels. The OCR drop observed after the injection of the ATP synthase inhibitor oligomycin reflected the ATP-linked respiration. The following injection of FCCP collapsed mitochondrial membrane potential and brought OCR to its maximum. Finally, the injection of the inhibitors of complex III and I antimycin A and rotenone, respectively, blocked the mitochondrial respiratory chain and strongly inhibited the respiration. The spare respiratory capacity was the difference between the maximal and the basal OCR values and measured the amount of extra ATP that could be produced by oxidative phosphorylation in the case of a rapid increase in energy demand or under metabolic stress condition.

The OCR and ECAR were expressed in pmol/min and mpH/min, respectively, and normalized to the µg of proteins of each sample.

### 4.6. Statistical Analysis

Statistical analyses were carried out using Graph- Pad Prism version 6.0 software (GraphPad Software, San Diego, CA, USA). Statistical significance of differences among data was estimated by Student’s *t* test or by analysis of variance (one-way ANOVA) followed by Tukey’s post-test, and *p* values are indicated where appropriate in the figures and in their legends. *p* values less than 0.05 were considered significant.

## Abbreviations

MPM	Malignant Pleural Mesothelioma
CDK	Cyclin-dependent kinase
RB	Retinoblastoma Protein
ECAR	extracellular acidification
OCR	oxygen consumption rate
